# Principles for building public-private partnerships to benefit food safety, nutrition, and health research

**DOI:** 10.1111/nure.12072

**Published:** 2013-10-11

**Authors:** Sylvia Rowe, Nick Alexander, Alison Kretser, Robert Steele, Molly Kretsch, Rhona Applebaum, Fergus Clydesdale, Deborah Cummins, Eric Hentges, Juan Navia, Ashley Jarvis, Ken Falci

**Affiliations:** SR Strategy LLCWashington, DC, USA; ILSI North AmericaWashington, DC, USA; Department of Food Science, Pennsylvania State UniversityUniversity Park, Pennsylvania, USA; Beltsville Human Nutrition Research Center, US Department of Agriculture, Agricultural Research ServiceBeltsville, Maryland, USA; Global Scientific and Regulatory Affairs, The Coca Cola CompanyAtlanta, Georgia, USA; University of Massachusetts at AmherstAmherst, Massachusetts, USA; American Academy of Orthopedic SurgeonsRosemont, Illinois, USA; McNeil Nutritionals, LLCFort Washington, Pennsylvania, USA; Kellogg CompanyBattle Creek, Michigan, USA

**Keywords:** conflict of interest, guiding principles, public-private partnerships, research

## Abstract

The present article articulates principles for effective public-private partnerships (PPPs) in scientific research. Recognizing that PPPs represent one approach for creating research collaborations and that there are other methods outside the scope of this article, PPPs can be useful in leveraging diverse expertise among government, academic, and industry researchers to address public health needs and questions concerned with nutrition, health, food science, and food and ingredient safety. A three-step process was used to identify the principles proposed herein: step 1) review of existing PPP guidelines, both in the peer-reviewed literature and at 16 disparate non-industry organizations; step 2) analysis of relevant successful or promising PPPs; and step 3) formal background interviews of 27 experienced, senior-level individuals from academia, government, industry, foundations, and non-governmental organizations. This process resulted in the articulation of 12 potential principles for establishing and managing successful research PPPs. The review of existing guidelines showed that guidelines for research partnerships currently reside largely within institutions rather than in the peer-reviewed literature. This article aims to introduce these principles into the literature to serve as a framework for dialogue and for future PPPs.

## Introduction

**Figure 1:**
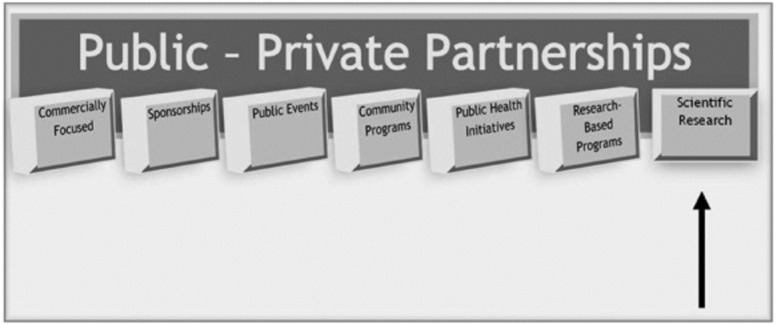
Types of public-private partnerships; this paper focuses on public-private partnerships in scientific research.

There are numerous types of public-private partnerships (PPPs), covering a spectrum from a purely research-driven partnership at one end to a commercially focused partnership at the other (Figure 1). This article focuses on PPPs to advance and promote scientific research to enhance public health in the fields of nutrition, health, food science, and food and ingredient safety. It is important to recognize that there are a wide variety of PPPs. For example, there are PPPs among community outreach organizations and both private and public sector entities. There are partnerships designed to promote or present public events, and to promote or administer public health initiatives. In addition, there are partnerships that amount to sponsorships, in which one public partner performs a public service of some kind and the private-sector partner provides financial and/or other resources, including but not limited to expertise, training, and access. It is important to stress that the present article examines issues around those collaborations pursuing scientific research on nutrition, health, food science, and food and ingredient safety, recognizing that not all research needs can be best addressed through PPPs.

## Methods

The present article builds upon historical precedence for the development of best PPP practices for scientific research. The following three-step process was used to distill a set of principles for the establishment and operation of research PPPs. 1) Sixteen US and global organizations currently or previously participating in PPPs were researched; of these, 14 had publically available information on PPP principles, best practices, etc., that were analyzed and synthesized (for a comprehensive list, see Appendix S1 in the Supporting Information online). 2) Four successful or promising PPPs focusing on the research areas of nutrition, health, food science, and food safety were studied in depth. Successful PPPs were defined as accomplishing defined goals established by the partners and promising PPPs were defined as moving towards defined goals established by the partners. 3) Twenty-seven background interviews were completed among experienced, senior-level individuals with financial and directorial responsibility for research, from academia, government, industry, foundations, and nongovernmental organizations (NGOs), on the challenges posed in creating PPPs and on recommendations for meeting those challenges to reach public health goals.

Interview subjects were selected based on their seniority and decision-making experience within their organizations; organizations were selected from a broad range of entities addressing research on food science and nutrition. Experts from 27 organizations were contacted and agreed to be interviewed under Chatham House Rules to promote candor. Six core questions were crafted to frame the interviews and probe knowledge of PPPs and potential PPP best practices (specific organizations chosen, areas of expertise, and core questions asked are included in Appendix S2, available in the Supporting Information online). To validate the findings, interview responses were then compared with data drawn from the existing PPPs that were studied and the literature review.

This article analyzes the following: 1) the evolving research environment and the possibility of combining public and private resources, in terms of broadening and enhancing cross-disciplinary expertise, and also to maximize financial resources; 2) the framework by which past and current PPPs have been guided; and 3) insights of the 27 interviewees. This work provides an overview of PPP development and offers a list of generalized potential best practices that were refined and tested against the operating principles of four existing, promising, or successful PPPs (Wheat and Barley Scab Initiative, Cocoa Genome Database, Feed the Future Initiative, and Research Integrity Roundtable) and against specific insights provided by the 27 interviewees. Finally, the authors articulate 12 principles, reflecting best practices, which are distilled from the paper's three-step process and offered here as a basis both for further discussion in the food and health research community and for possible implementation.

## Historical Perspective: The Rationale for Public-Private Partnerships

One key reason offered for establishing PPPs is to create a collaborative environment to maximize cross-disciplinary expertise among government, academic, and industry researchers – an enhancement that could, in turn, help solve complex research problems. Other possible benefits include maximizing resources and sharing risks across partner organizations. Clearly, however, organizers must address a number of issues when merging public and private interests in a common partnership, the resolution of which might well yield a set of general principles to guide in future PPP endeavors. These principles would address such issues as establishing public health goals, progress/success monitoring, scientific metrics, partner roles/accountability and work assignments, membership equality, power balance, conflicts of interest and research objectivity, partner compatibility, partner sharing and commitment, academic/civil society inclusion, flexibility and transparency, and consideration of a third-party convener to ensure rule compliance. These issues are addressed in greater detail in the research section of this work.

Data from the US Department of Agriculture (USDA) show a flattening or declining trend in funds available for agricultural research over the past several years, as well as a corresponding increase in the portion of funding provided by the private sector.^1^ Some experts have noted the “reconfiguration of the international health landscape” through the growing number and influence of global PPPs concerned with public health.^2^ The National Institutes of Health (NIH) Common Fund has emphatically endorsed the concept and practice of building collaborations involving both public and private sectors in the conduct of scientific research, stating that “partnerships between government agencies and private industry already have extended and accelerated NIH research, research training and the dissemination of information in diverse and creative ways.”^3^ A quantitative review of funding trends published by Dorsey et al. in the *Journal of the American Medical Association* concluded that “after a decade of doubling, the rate of increase in biomedical research funding slowed from 2003 to 2007, and after adjustment for inflation, the absolute level of funding from the National Institutes of Health and industry appears to have decreased by 2% in 2008.”^4^ Internationally, the Canadian government is currently moving forward in providing funds for collaborative research projects partnering industry with the academic community. The Canadian Institutes of Health Research has made any Canadian university eligible to receive funding after securing matching funds from an industry partner.^5^

The US government recently stressed the need for greater synergy among the various scientific disciplines in the area of basic biological research. For example, the National Bioeconomy Blueprint initiative announced by the Obama Administration in April 2012 offers a rationale for and describes the funding benefits of broadening research collaborations as follows: “The complexity of modern research questions requires that traditional boundaries between fields of study become permeable and programs concentrate expertise from diverse disciplines around societal challenges where it is needed most… An increased focus on entrepreneurship, translational sciences, regulatory science, and technology transfer can help ensure that ideas with potential for application move beyond the laboratory… Federal agencies should focus on building new, and augmenting existing, stakeholder collaborations to inform efforts, streamline processes, and reduce costs and response times, while preserving safety and ensuring substantive benefit to public health.”^6^

Partly for the reasons offered here, more than a few experts have urged greater involvement of the private sector, particularly through PPPs, in the funding of projects critical to public health. They believe that leveraging the proficiencies and competencies of as broad a range of stakeholder experts as possible could maximize available expertise, collectively achieving the best research outcomes while extending and enhancing limited financial and other resources.

## Research: Defining Best Practices and Ethical Underpinnings of Effective PPPs

Although the published literature is relatively sparse on PPPs devoted to nutrition and food research, the most recent comprehensive work in this area was provided by a workshop of The National Academies, as outlined in a summary available online.^7^ A key discovery of the present article is that much of the recent work on the issue of food and nutrition research partnerships resides institutionally in organizations such as The National Academies, the World Health Organization, NIH, USDA, and the Institute of Medicine, rather than in the published peer-reviewed literature.

There is, however, some published work in the field of agricultural research. In an article published in the *Journal of Technology Transfer*, Spielman and Grebmer hypothesized that the potential offered by PPPs in agriculture is “constrained by the fundamentally different incentive structures” in public and private organizations.^8^ In addition, King et al. made the following point in a USDA Economic Research Service Economic Brief: “While the use of CRADAs (Cooperative Research and Development Agreements), patenting, and other technology transfer instruments could potentially divert public research away from its central mission, such arrangements may also draw private capital into areas that serve important societal needs but where market failures are most evident, like human nutrition.”^9^

The peer-reviewed record on medical research provides a glimpse into the challenges and promise of PPPs. In Europe, a public-private initiative is under way to speed health solutions to the public by engaging the pharmaceutical industry. However, as Tachibana pointed out in an article in *Science* referencing the European Innovative Medicines Initiative, “Industry-academic collaborations are like partners skilled in different dances trying to reach a compromise between waltz and salsa. Rhythms, pace, and expected outcomes can be frustratingly at odds, as university researchers prioritize education and basic research and corporate scientists pursue products and profits. Success depends on finding common goals and negotiating plans that pay off financially and intellectually for all parties.”^10^

In addition, Buse and Walt argued the following in an article on emerging global PPPs published in the *World Health Organization Bulletin*: “However, while there are many positive aspects to these new global PPPs, there is also a great deal of uncertainty and some cause for concern. We have argued that public and private sectors are driven by differing ethos and principles, but how these unique attributes will be affected by partnerships remains to be seen.”^2^

It is clearly only through a well-articulated set of guiding principles that such concerns may be addressed. A review of a number of existing and past PPPs yields a tentative list of likely best practices and ethical considerations in creating and managing such sustainable collaborations. Commonalities and key principles discovered, with the specific researched partnerships stressing them, are captured in this section. Additional details on the organizations and examples described herein are available in Appendix S1 in the Supporting Information online.

### Commonalities and key principles

#### Leveraging of partners' capabilities for the health of the public

The White House Office of Social Innovation and Civic Participation stresses that the “core competencies” of external stakeholders should be enlisted to “leverage collective action” by involving as broad a spectrum of actors as possible.^11^ A White House policy review suggested that in effective PPPs, pragmatism rather than ideology should govern, the various partners' “value propositions” should be fully appreciated and plans jointly developed, existing private-sector mechanisms ought to be fully utilized, and a mechanism for evaluating effectiveness should be inherent to the design of partnerships.^12^

#### A good fit

The Commonwealth Scientific and Industrial Research Organisation (CSIRO) stresses an examination of such criteria as to whether there is an appropriate fit, noting that it seeks partners with complementary capability, capacity, resource access, and experience. CSIRO further stresses a belief that partners should bear risk appropriate to their contribution and share fairly in the benefits from the research based on the value they bring to the partnership.^13^

#### Accountability and transparency

The Canadian Institutes of Health Research emphasizes accountability and transparency among its partners and between its partnerships and the public, as well as urging “… open, 2-way communication and dialogue among partners to achieve positive results desired by each Partner, to solve problems as early as possible, and to build trust.”^14^

#### Fair, unbiased project selection and disclosure of interests

The NIH stresses that the research project selection process with partners be “fair, unbiased, and as transparent as possible with reasonable opportunity for input by all materially affected stakeholders.” The NIH further requires that any of its partner executive or steering committee members “having a commercial interest in a specific project should disclose the existence and nature of the interest or recuse himself or herself from voting.”^15^

#### Honest interactive communication among partners and between partnerships and the public

The Building Trust Initiative of the Canadian Obesity Network urges an ongoing dialogue between the public and the private-sector funders, which it calls “honest interactive communication that enables common understanding.” The network further advises that partnership-enabling factors include the following: a willingness to take risks and accept that outcomes may not always be perfect, a willingness to make compromises, and a willingness to admit to weaknesses, personal, organizational, or within a sector.^16^

#### Clearly understood and agreed-upon objectives

The European Commission, in its guidance for public- and private-sector collaborations, stresses that public entities need to be realistic about the skills and experience they have to bring to partnerships and advises that they “integrate private sector expertise if required.” The European Commission further advises that “all parties must recognise and understand their (separate) objectives…”^17^

#### Public benefit from intellectual property generated by partnership

The Global Alliance for Improved Nutrition, recognizing that its efforts will likely generate knowledge, data, and public health research, supports “appropriate protection and use of intellectual property where this will maximize … global health goals.”^18^

#### Mutual trust and cooperation

The Pacific Economic Cooperation Council recommends that in creating cross-sector partnerships, the public- and private-sector members have a clear understanding of their distinct roles and abilities in the collaboration. High on its list of partnership best practices are mutual trust and cooperation, transparency of procedures, performance criteria, and review mechanisms.^19^

#### Broad-based partner participation including consumer perspectives

The Reagan-Udall Foundation, created by the US Congress to promote PPPs to support the US Food and Drug Administration (FDA), embraces core principles to spearhead complex research collaborations involving public and private partners, ensure broad-based participation (including consumer perspectives), and ensure that new knowledge gained from the collaboration is in the public domain.^20^

#### Strategic and financial long-term commitment of partners

The National Collaborative on Childhood Obesity Research regards its strategic mission as long term, with consequent binding stipulations on prospective members, requiring that they also regard its strategic mission as long term. It also requires collaborators to commit to funding and participation in the partnership's other activities, meetings, and projects, as they arise in the future.^21^

#### Identifying and managing potential legal/ethical issues

The FDA, in its “steps for developing leveraged projects,” recommends identifying “potential legal and ethical issues relevant to the proposed activities, potential collaborators or the funding or contract mechanism(s) being considered.” The FDA also urges specification of funding arrangements, activities to be pursued and activities not to be pursued, as well as points of communication and coordination.^22^

## Analysis of Identified PPPs

The following analysis generalizes from four existing PPPs in order to distill some likely additional best practices, and to test against principles already captured, for future research-focused collaborative efforts.

### Wheat and Barley Scab Initiative

The US Wheat and Barley Scab Initiative (USWBSI) is an effective PPP that has emphasized multidisciplinary approaches. The USWBSI was organized to control the *Fusarium* head blight, also known as wheat and barley scab, which emerged in the 1990s as a serious and costly threat to US agriculture. The initiative, which was spearheaded by the USDA Agricultural Research Service, recruited industry scientists along with academic and government researchers as well as others. The USWBSI is guided by a steering committee composed of growers, farm organizations, food processors (e.g., millers, bakers, pasta manufacturers, and brewers), scientists (from Land Grant universities, the USDA, and private companies), and consumer groups. According to the initiative, “The speed and magnitude of the success the industries and organizations involved have had in generating funds and associated research plans is an arguably unprecedented happening in U.S. plant agriculture.”^23^

This PPP's effectiveness is based on its longevity (a 15-year record of progress) and its enormous multidisciplinary base of expertise drawn from food producers, growers, scientists, and consumers. The potential best practice to be taken from this PPP is the conscious enlistment of researchers from a variety of disciplines, notably including partners with practical, real-world experience with the plant blight.

### Cacao Genome Database

This partnership consists of public and private members, including the USDA Agricultural Research Service, IBM, Mars Inc, the National Center for Genome Resources, Clemson University, Indiana University, Washington State University, and the Hudson/Alpha Institute for Biotechnology, which collaborated in tackling the task of sequencing the cacao genome. Features of the collaboration are that it is an atypical partnership across industry sectors and that its activities take place in precompetitive space with its results fully available to the public. The objective is to offer scientific resources for the agricultural improvement of a crop that provides economic livelihood for more than 6.5 million farmers in Africa. A comment on the modus operandi for the project's collaborative efforts states the following: “Most importantly, we were able to benefit from significant advances in sequencing technology over the past two years … We had a methodology that was trackable to everyone at all times. This coalition of like-minded scientists worked tirelessly to bring this invaluable resource to fruition and make it available to the research community in a timely manner.”^24^

The Cacao Genome Database project appears to be a successful PPP for two standout accomplishments. First, it has brought together disparate and uniquely qualified industry partners, one well versed in the agricultural and nutritional issues around cacao and one well versed in the data technology necessary for the sequencing. Second, the partnership has committed itself at the outset to releasing the ultimate data into the public domain. The potential best practices from this research effort include a systematized communication/feedback plan involving all partners, along with a method for tracking progress among the various scientists and researchers from both the public and private sectors.

### Feed the Future Initiative

Another agricultural partnership that strives to promote health internationally is the Feed the Future Initiative, through which the US government initially pledged $3.5 billion over three years to address and ameliorate the global spike in food prices that occurred in 2007–2008. According to one of the several government websites devoted to the partnership: “The U.S. Government's Feed the Future Initiative utilizes innovation, research, and development to improve agricultural productivity, link farmers to local and regional markets, enhance nutrition, and build safety nets. These investments will increase the supply of food where it is needed and help vulnerable people withstand price shocks better.”^25^

The collaboration includes the governments of more than a dozen other nations to speed agricultural technology to alleviate global hunger and to finance food security strategies in the world's poorest countries. In addition to foreign governments, partners include a number of US federal agencies, foundations, international nonprofit organizations, and members of the global food and agricultural industry.^26^

Although its central promise has not yet been fulfilled, one of Feed the Future's key qualities is its long-term commitment to achieving its goals and to the use of “benchmarks and targets to measure progress toward shared goals.” Its sustained accomplishments clearly rest upon the many governmental and private-sector partners supporting its multitargeted food security/nutrition efforts. The best practices to draw from this example include not only the key metrics to track possible success, but also the PPP's extremely broad perspective, partner acceptance of very great financial needs, and long-term commitment to global health.

### Research Integrity Roundtable

The Keystone Center's Research Integrity Roundtable is a relatively recent US partnership devoted to food and health research. The partnership's purpose is to anticipate ethical and conflict-of-interest issues around scientific research and to manage them to ensure scientific integrity and public and regulatory trust in science. The center states the following: “Participants – drawn from industry, advocacy, academia, and key Federal agencies – will work to identify or develop better procedures, policies, and protocols to help address concerns about conflicts of interest or bias in a variety of manifestations involving the integrity of data (and the processes that produced it) and of scientists and their opinions.”^27^

The Keystone Center is itself a public-private collaboration whose sustainability is attested to by its 40-year record and timely commitment to addressing society's major challenges, as demonstrated in its work on a 2012 HBO documentary on obesity. It would appear that the keys to this organization's effective partnership are its focus on communication of scientific research to the broader public, thereby enhancing public understanding of, and credence in, science, and its embracement of an explicitly articulated shared value system that stresses independence, impact, inclusiveness, inquiry, and innovation.

## Interview Results: Drawing on Experience

Interviews were conducted with 27 experts in regard to forging a single research-focused organization from public and private contributors. These individuals, with financial and directorial responsibility for research in government agencies, NGOs, industry, foundations, and academia, and including ethicists, offered their guidance and/or warnings about partnering public and private interests related to scientific research on food and health.

The discovery from the interviews was a validation of the criteria listed herein, based on the analysis of the organizations listed in Appendix S1 in the Supporting Information; the findings highlighted that PPPs should have clearly identified, accomplishable goals to improve public health, and progress toward those goals must be measurable and trackable. In addition, participants should be of roughly equal decision-making authority, they should effectively manage any conflicts of interest, and above all, they should be flexible, willing to adapt and evolve, and have mutual trust.

### Addressing the challenges

The experts interviewed for this work offered a variety of supporting, and in some cases critical, comments regarding their experiences and expectations. In general, the interviews identified a number of themes that demonstrate the diverse views of PPPs, as well as potential areas in which acceptance of the PPP might be problematic. Issues for consideration were as follows: a shared goal for the common good, transparency of action, global societal issues relevant to all stakeholders, recognition of partners' roles and ensuring appropriate representation, attitudes and perceptions concerning potential bias or self-interest, appropriate boundaries for investors in the research, and achievement of mutual respect and trust in the partnership process. (See Appendix S2 in the Supporting Information for a list of the interviewees' organizations and a selection of salient quotes.)

There may be deal breakers in trying to forge a partnership among public and private-sector players, such as contractual provisions in the partnership agreement that amount to structural barriers to effectiveness or credibility. These deal breakers may be so destructive to a sense of balance among the various partners as to work against any chance of a successful long-term research enterprise. Among these might be clauses written into the partnership charter that, in effect, hand over control to one partner at the expense of the other partner's interests. Clearly, it is of crucial importance for prospective partners to address potential deal breakers at the initial stages of PPP formation, which can be quite protracted.

The overall interview feedback suggests that research-driven organizations will, out of necessity and with the potential to benefit the health of the public, continue to seek partnerships including private-sector stakeholders. As one government agency official stated, “Public–private partnerships will be more essential in the future for two reasons: science is becoming transdisciplinary and costs are prohibitive. No one sector can address issues alone (for example, even the pharmaceutical and academic sectors are working collaboratively on basic biomedical research). The challenge is how best to do it.”

### Major points of agreement and caveats

Analysis of the interviews revealed broad support for the inclusion of private-sector researchers in publicly focused partnerships, some caveats were identified. An Institute of Medicine official who has worked on prioritizing criteria for research projects stressed that before setting out to establish a PPP, as much preliminary work as possible should be done, including groundwork on the goals and objectives of the research, conflict issues among the partners, funding issues, and so forth. It is possible that several years of preparation may be necessary before a collaboration can be formally launched.

The interviews also revealed reasonably strong agreement that a nutrition research partnership, for a variety of reasons, might be difficult to craft. However, a PPP devoted to food safety research would be more realistic and relatively straightforward because food safety is a universally accepted goal with collective responsibility. There was agreement that the availability of research funds would be a major driver in the creation of PPPs and that a diversity of partners, including those engaged in for-profit enterprise, would offer a potential net benefit to public health. However, concerns were expressed even by some experts who strongly favor bringing together public and private-sector scientists; one issue cited was the differing perspectives between public and private entities in terms of time horizons and expected results.

Virtually all of the individuals interviewed expressed concerns about conflicts of interest related to private industry (e.g., the food industry, for the purposes of this work). They offered the following strategies for alleviating their concerns: blinding of funding sources; allowing ultimate control of the research design, interpretation of the findings, and so forth to rest with a public (nonconflicted) entity; including a multiplicity of private-sector interests in the partnership to mitigate the influence of any particular one; and securing oversight by a nonconflicted third party. Other challenging issues include intellectual property, proprietary information, and global societal issues such as population growth, food security, climate change, and critical nutrition and food safety issues.

## Working in Harmony toward a Framework

There have, of course, been numerous other organizational initiatives around constructing a generalized set of principles for PPPs, such as the Centers for Disease Control and Prevention Foundation's partnership policies for health initiatives^28^ and various international economic partnerships.^29^ However, as previously noted, relatively little has been offered in the peer-reviewed literature in the way of generalized food and nutrition health research-related partnership guidance. Two recent and notable attempts at formalizing best practices and the process for establishing PPPs in the area of food research were promoted by the Institute of Medicine Food Forum: the Leveraging Food Technology for Obesity Prevention and Reduction Efforts workshop held in November 2010 and the Building Public–Private Partnerships in Food and Nutrition workshop held in November 2011.^30^–^31^

An NIH/USDA April 2012 workshop yielded a document, still in the draft stage, titled “Nutrition Translation from Bench to Food Supply – Matrix of Prioritization Criteria for Research Questions” (NIH/USDA, unpublished data). This work is an attempt at generalizing guidance for food and health research PPPs in the future (see Appendix S3 in the Supporting Information online for a summary). The document offers the following suggested questions to ask before establishing a broad PPP for food research: Does the research have the potential for significant public health impact? Does it have implications across many different food and/or beverage categories? Is it precompetitive in nature and what does this mean for food industry?

As for prospective partners, the NIH/USDA draft document recommends, in part, that each party has clearly explained the resources that it can contribute and the goals it hopes to achieve through the research. The document also recommends that the prospective partnership facilitate advancement in the proposed specific area of research or in a particular research question in a manner that would not be likely with individual entities working independently, or with a partnership between a single company and a single government agency (NIH/USDA, Appendix S3).

A US Congressional subcommittee pointed to an ongoing funding shortfall in 2007 when it proposed the Reagan-Udall Foundation for the FDA, noting at the time that “science at the FDA is in a precarious position: the Agency suffers from serious scientific deficiencies and is not positioned to meet current or emerging regulatory responsibilities.” More specifically, the subcommittee observed that although demands on the FDA had increased due to ongoing scientific innovation, resources had not kept up with these demands and the demands had exceeded the FDA's regulatory scientific capacity.^20^ The same argument that the demand for resources has not kept up with the pace of and need for scientific innovation can be made for other scientific research, especially in recent economic times.

## Conclusion

The aim of the work presented here was to determine whether a list of general principles can be established concerning the creation and management of PPPs. After researching a number of existing PPP guidelines both in the peer-reviewed literature and internally at non-industry organizations and successful or promising PPPs, and interviewing 27 partnership-experienced individuals from academia, government, NGOs, foundations, and industry, a comprehensive list was assembled of best practices that are common to most research PPPs. Following are 12 proposed principles, offered in no strict chronological order because each PPP has unique characteristics.

PPPs should do the following:

1. Have a clearly defined and achievable goal to improve the health of the public
2. Articulate a clear statement of work, rules, and partner roles, responsibilities, and accountability, to build in trust, transparency, and mutual respect as core operating principles – acknowledging there may be “deal breakers” precluding the formation of an effective partnership in the first place
3. Ensure that objectives will meet stakeholder partners' needs, with a clearly defined baseline to monitor progress and measure success
4. Considering the importance of balance, ensure that all members possess appropriate levels of bargaining power
5. Minimize conflict of interest by recruiting a sufficient number of partners to mitigate influence by any single member and to broaden private-sector perspectives and expertise
6. Engage partners who agree upon specific and fundable research question(s) to be addressed by the partnership
7. Enlist partners who are committed to the long-term goals as well as to the sharing of funding and research data
8. Along with government and the private sector, include academics and other members of civil society as partners
9. Select objective scientific measurements capable of providing common ground for both public- and private-sector research goals
10. Adopt research questions and methodologies established by partners with no vested financial interest in them, ideally in the precompetitive space
11. Be flexible and ensure ongoing transparent communications
12. Consider a third-party convener to ensure equality at the table, clarify rules, establish operational guidelines, and specify funding arrangements.

It seems clear that the complex business of creating a wide-ranging collaborative effort to have a significant positive impact on public health is not a one-size-fits-all proposition and requires a comprehensive commitment from preliminary work through completed research. However, given the broad agreement cited here, it also seems clear that the effort to create general principles is likely to be helpful. The realities confronting science and public health make this imperative.

Indeed, in announcing findings from its June 2012 study on “Research Universities and the Future of America: Ten Breakthrough Actions Vital to Our Nation's Prosperity and Security,” the National Research Council called on both public and private sectors to do more to ensure adequate and stable research funding over the coming decade. The National Research Council study committee “has taken stock of the health of our nation's research universities today and envisioned the role we would like them to play in our nation's life 10 to 20 years from now.” One of its conclusions states the following: “Businesses, which have long relied on research universities for talent and technology, should also play a bigger part in ensuring their health. … Federal and state policies should encourage collaboration between U.S. national laboratories, businesses, and universities in order to enable large-scale, sustained research projects.”^1^

The hope is that, at a minimum, there will be further critical engagement on the presently proposed principles among key public and private stakeholders going forward.

## Notes

Appendix S1 Organizations with principles on publicprivate partnerships

Appendix S2 Summary of interview process, including organizations represented, questions asked, and representative comments from interviewees.

Appendix S3 Summary of NIH/USDA workshop “Nutrition Translation from Bench to Food Supply-Matrix of Prioritization Criteria for Research Questions”
