# Supplementary material for: Principles for building public-private partnerships to benefit food safety, nutrition, and health research
Source: Nutr Rev. 2013 Oct 11;71(10):682–91. doi: 10.1111/nure.12072 (PMC3886300; doi:10.1111/nure.12072)
Supplement: Appendix S2 — Summary of interview process, including organizations represented, questions asked, and representative comments from interviewees. [file nure0071-0682-sd2.docx]

Appendix S2

Organizations and Representative Comments from Interviewees

Organizations:

Consumers Union

Food and Drug Administration

Foundation for the National Institutes of Health

International Life Sciences Institute (ILSI) Center for Risk Science Innovation and Application

International Life Sciences Institute (ILSI) SE Asia

Institute of Medicine

Mars, Inc.

National Academy of Sciences

National Institutes of Health National Cancer Institute

Pennsylvania State University

The Keystone Center

The PEW Charitable Trusts

Unilever

University of Ottawa

University of Pennsylvania

U.S. Department of Agriculture

U.S. Department of Health and Human Services

World Health Organization

Interviewees were asked a number of questions designed to elicit their views of the strengths/challenges of PPPs and of what they considered the greatest potential benefits of PPP formation. They responded as individuals, rather than as representatives of their organizations. The substance of the questions included:

1. View on PPPs – what is your general view on partnerships between the private sector and public sector, to reach public health goals.
2. Guidelines – a number of organizations, associations and collaborations have developed, vetted and shared guidelines on how to put in place effective, responsible and beneficial PPPs.  In your view, what are the key elements of a PPP that must be adhered to by either or both parties?
3. Can you provide us with examples of PPPs that you believe were successfully implemented whereby both partners would call the partnership a success?  Please provide as many as you are aware of and explain why you believe they were a success?  What were the elements of best practices in these examples?
4. Can you provide examples of PPPs that you believe were not as successful or not successful for one or both of the partners and explain why?  Please provide as many for which you are aware.
5. Case studies – in addition to your responses to #3and #4, can you provide any other case studies that you suggest we look at?
6. International View – PPPs are developing in the US and Canada.  Can you share any examples or learning you have observed from other nations?

Some specific, salient observations made by the surveyed individuals in support of PPPs were:

- From a university ethics professor: “The critical element is the shared goal that enables the private sector to put aside institutional norms for the public good. If not articulated, vetted, and transparent, it will be perceived as industry’s attempt to ‘co-opt’ government agencies.”
- From an NGO official: “Public–private partnerships work best when there is a wide array of interests with a seat at the table that don’t operate behind closed doors.”
- From a government official: “Major global societal issues such as population growth, food security, and climate change need to be taken account of in building partnerships, and these societal challenges require that the private sector be involved as these will affect their business.”
- From an industry scientist: “Nutrition questions are complex and address diet and dietary factors. Scientists within industry have a better understanding of food and the food supply than academics, but the combined experience is invaluable.”

Among the comments and suggestions uncovered in the survey, there were some pointed reservations that were more or less altogether critical of the utility of PPPs. A sampling of these comments follows:

- From a former NGO official: “A roadblock (to the usefulness of PPPs) is the perception that they are pursued to give credibility to business interests.”
- From a physician in obesity practice: “There are no good mechanisms to create a barrier between those experts determining research methodology/design and the industry funder if the funding is transparent to the experts.”
- From a university ethicist: “(Public–private partnerships have) the potential to corrupt the fidelity/integrity of the public institution and divert its priorities from its mission; and (they risk) the destruction of public trust in the public institution.”
- From an ethicist: “There can be no public trust if there is any industry funding of research, hence a public–private partnership is not a worthwhile model, even if through shared resources and within a credible operational framework.”
