# Supplementary material for: Principles for building public-private partnerships to benefit food safety, nutrition, and health research
Source: Nutr Rev. 2013 Oct 11;71(10):682–91. doi: 10.1111/nure.12072 (PMC3886300; doi:10.1111/nure.12072)
Supplement: Appendix S3 — Summary of NIH/USDA workshop “Nutrition Translation from Bench to Food Supply-Matrix of Prioritization Criteria for Research Questions” [file nure0071-0682-sd3.docx]

**Appendix S3—Nutrition Translation from Bench to Food Supply**

*DRAFT Matrix of Prioritization Criteria for Research Questions*

|  | **DEAL BREAKERS** | **RATING SCALE** | | | **Rating** |
| --- | --- | --- | --- | --- | --- |
|  |  | **0** | **1** | **2** |  |
| 1 | **Public Health Impact**  Will the advancement of research in this area/on this question via a partnership have the potential for a significant public health impact? | The public health impact of the research is neutral or unknown at this time. | The research will positively impact one or more aspects of public health, but the effects would be difficult to quantify. | The research will positively impact multiple aspects of public health, which can be quantified and measured for evaluation. |  |
| 2 | **Increased Effectiveness**  Will the partnership facilitate advancement in this area of research or this particular research question in a manner that is not likely with individual entities working independently? | There is no anticipated mutual benefit of exploring this research question/area with a partner organization. | The research question/area is best addressed through a single company and single government agency partnering. | The research area/question has implications across many different food and/or beverage categories. |  |
| 3 | **Precompetitive Space***  Is the research question/area pre-competitive in nature?   - - *Need to define what “precompetitive” means for the food industry*   *rating scale is based on the Technology Readiness Level measure utilized by many government agencies and private industries (see appendix) | The research would result in product(s) or technology that would be ready for testing or translation. | The research is more applied and may lead to the development of a proof of concept. | The research would query basic scientific experimentation (e.g., bench) or natural experiments followed-up with validation studies. |  |
| 4 | **Risks and Benefits**  Do the benefits add value and outweigh the risks for a partnership?   - Conflicts of interest - Opportunity costs of distraction from engaging in activities which are not of interest to a partner, but may be important for public health and nutrition goals - Loss of legitimacy with key allies (shareholders, funders, constituents) - Funding driven shifts in policy - No guarantee that the partnership will stop a partner from engaging in activities that are counter to the overall objective - Reduced sales | The costs and risks exceed the benefits. | There are known risks, but they can appropriately be mitigated and managed, so that the benefits may be seen. | The benefits easily outweigh the risks. |  |
| 5 | **Regulatory Implications**  What is the regulatory plausibility of success? | Federal regulatory frameworks do not currently support the application/ translation of the research. | Some opportunities exist within current regulations to encourage translation to the food supply but challenges are anticipated. | No regulatory barriers known. |  |
|  | **ENGAGEMENT DECISIONS** | **RATING SCALE** | | | **Rating** |
|  |  | **0** | **1** | **2** |  |
| 6 | Can the partnership be defined with clear objectives and outcomes? |  |  |  |  |
| 7 | Has each party clearly explained the resources that they can contribute? |  |  |  |  |
| 8 | Has each party clearly explained the goals that they hope to achieve through the research? |  |  |  |  |
| 9 | Does each party bring resources, skills, knowledge, and/or capabilities to the initiative? |  |  |  |  |
| 10 | Does the execution of the initiatives support/further the mission of each partner? |  |  |  |  |
| 11 | Are arrangements for handling pre-existing IP and managing new IP feasible? |  |  |  |  |

**Appendix S3, continued**

**TECHNOLOGY READINESS LEVEL (TRL) DEFINITIONS**

*Taken from Department of Defense and Department of Energy*

| **Technology Readiness Level** | **Description** |
| --- | --- |
| 1. Basic principles observed and reported | Scientific research begins translation to applied R&D - Lowest level of technology readiness. |
| 2. Technology concept and/or application formulated | Invention begins - Once basic principles are observed, practical applications can be invented. Applications are speculative and there may be no proof or detailed analysis to support the assumptions. Examples are limited to analytic studies. |
| 3. Analytical and experimental critical function and/or characteristic proof of concept | Active R&D is initiated. This includes analytical studies and laboratory studies to physically validate the analytical predictions of separate elements of the technology. Examples include components that are not yet integrated or representative. |
| 4. Component and/or breadboard validation in laboratory environment | Basic technological components are integrated to establish that they will work together. |
| 5. Component and/or breadboard validation in relevant environment | Fidelity of breadboard technology increases significantly. The basic technological components are integrated with reasonably realistic supporting elements so they can be tested in a simulated environment. Examples include “high-fidelity” laboratory integration of components. |
| 6. System/subsystem model or prototype demonstration in a relevant environment | Representative model or prototype system, which is well beyond that of TRL 5, is tested in a relevant environment. Represents a major step up in a technology’s demonstrated readiness. Examples include testing a prototype in a high-fidelity laboratory environment or in a simulated operational environment. |
| 7. System prototype demonstration in an operational environment. | Prototype near or at planned operational system. Represents a major step up from TRL 6 by requiring demonstration of an actual system prototype in an operational environment. |
| 8. Actual system completed and qualified through test and demonstration. | Technology has been proven to work in its final form and under expected conditions. In almost all cases, this TRL represents the end of true system development. Examples include developmental test and evaluation of the system in its intended environment. |
| 9. Actual system proven through successful operations. | Actual application of the technology in its final form and proven through successful operations. |

**First draft of prioritization questions**

The Research

- Has the potential for significant public health impact.
- Has implications across many different food and/or beverage categories.
- Is pre-competitive in nature.
  - What does this mean for food industry?
  - Is this realistic with biomarker development? Perhaps because foods are not patentable and substantiated claims can be used by any company, a cooperative approach to biomarker identification would more appropriately allow for shared investment in the process.
- Bridges translation from bench to food supply.
  - Can this be true with bullet above?
- Addresses a problem for which a solution could feasibly be provided via the food supply.

The Partners

- Each party has clearly explained the resources that they can contribute and the goals that they hope to achieve through the research.
- Each party brings resources, skills, knowledge, and/or capabilities to the initiative.
- Each party will further its mission through execution of the initiative.
- Partnership will facilitate advancement in this area of research or this particular research question in a manner that is not likely with individual entities working independently, or with a single company and single government agency partnering.
  - More rapid advancement?
  - High cost shared among many parties?
  - Complementary resources?
  - Other?
